# 551. A Multistate Investigation of Serious Adverse Events, Including Deaths, Following Ceftriaxone Injections, September 2024–June 2025

**DOI:** 10.1093/ofid/ofaf695.024

**Published:** 2026-01-11

**Authors:** Rebecca Pierce, Radhika Agarwal, Dumbani Kayira, Jennifer Lind Lyles, Maribeth Sivilus, Melissa A Morrison, Andrew W Stubblefield, Melanie Roderick, Karen Landers, Scott Harris, Theresa Dulski, Kelley Garner, Rachana Bhattarai, Juliet Stoltey, Lauren Biehle, Christopher A Czaja, Joshua T Hoffner, Argentina Charles, JoAnna Wagner, Trent Gulley, Haley Beeman, Douglas A Thoroughman, Michael J Curran, Andrea H Flinchum, Andrea Salinas, Grace Lee, Riley Moore, Brenda M Brennan, Gargi Patel, Jason Mehr, Dat Tran, Liz Breitenstein, Jenna N Sinkevitch, Stacy Tressler, Cara Bicking Kinsey, Marco Tori, Christopher Wilson, Simone Godwin, Malakai Miller, Cullen Adre, Shehzana Hussaini, Carolyn Hembree, Allison G Lafferty, Shaina Bernard, Shannon Ruelle, Mark P Loh, B S Biochemistry, Kimberly Garner, Maya Beganovic, Ramesh GopalaSwamy, Kiran M Perkins

**Affiliations:** Centers for Disease Control and Prevention, Atlanta, Georgia; Centers for Disease Control and Prevention, Atlanta, Georgia; U.S. Centers for Disease Control and Prevention (CDC), Atlanta, Georgia; Centers for Disease Control and Prevention, Atlanta, Georgia; Centers for Disease Control and Prevention, Atlanta, Georgia; Centers for Disease Control and Prevention, Atlanta, Georgia; Alabama Department of Public Health, Decatur, Alabama; Alabama Department of Public Health, Decatur, Alabama; Alabama Department of Public Health, Decatur, Alabama; Alabama Department of Public Health (ADPH), Montgomery, Alabama; Centers for Disease Control and Prevention, Atlanta, Georgia; Arkansas Department of Health, Little Rock, Arkansas; Arizona Department of Health Services, Phoenix, Arizona; California Department of Public Health, Richmond, California; Colorado Department of Public Health and Environment, Denver, Colorado; Colorado Department of Public Health and Environment, Denver, Colorado; Connecticut Department of Public Health, Hartford, Connecticut; Florida Department of Health, Tallahassee, Florida; Georgia Department of Public Health, Atlanta, Georgia; Indiana Department of Health, Indianapolis, Indiana; Indiana Department of Health, Indianapolis, Indiana; US Centers for Disease Control and Prevention, Frankfort, Kentucky; Kentucky Department for Public Health, Frankfort, Kentucky; Kentucky Department for Public Health, Frankfort, Kentucky; Louisiana Department of Health, New Orleans, Louisiana; Louisiana Office of Public Health, New Orleans, Louisiana; Michigan Department of Health & Human Services, Lansing, Michigan; Michigan Department of Health and Human Services, Lansing, Michigan; New Jersey Department of Health, Infectious and Zoonotic Diseases Department, Trenton, NewJersey; New Jersey Department of Health, Trenton, NewJersey; Oregon Health Authority, Portland, Oregon; Oregon Health Authority, Portland, Oregon; Pennsylvania Department of Health, Harrisburg, PA, Pennsylvania; Pennsylvania Department of Health, Harrisburg, PA, Pennsylvania; Bureau of Epidemiology, Pennsylvania Department of Health, Harrisburg, Pennsylvania; Centers for Disease Control and Prevention/ South Carolina Department of Public Health, Columbia, South Carolina; Tennessee Department of Health, Nashville, Tennessee; Tennessee Department of Health, Nashville, Tennessee; Tennessee Department of Health, Nashville, Tennessee; Tennessee Department of Health, Nashville, Tennessee; Harris County Public Health, League City, Texas; Harris County Public Health, League City, Texas; Vermont Department Of Health, Waterbury, Vermont; Virginia Department of Health, Richmond, Virginia; Food & Drug Administration, Irvine, California; U.S. Food and Drug Administration, Irvine, California; U.S. Food and Drug Administration, Irvine, California; U.S. Food and Drug Administration, Irvine, California; US Food and Drug Administration, Silver Spring, Maryland; Centers for Disease Control and Prevention, Atlanta, Georgia

## Abstract

Wednesday, October 22, 2025: 11:30 AM

**Background**: Serious adverse events (SAEs) related to ceftriaxone, a widely used cephalosporin antibiotic, are considered rare. After a 2024 SAE cluster in Alabama, the Centers for Disease Control and Prevention (CDC), with state/local partners and the US Food and Drug Administration (FDA), conducted a nationwide investigation of SAEs, including deaths, among patients who received ceftriaxone.

**Table.**
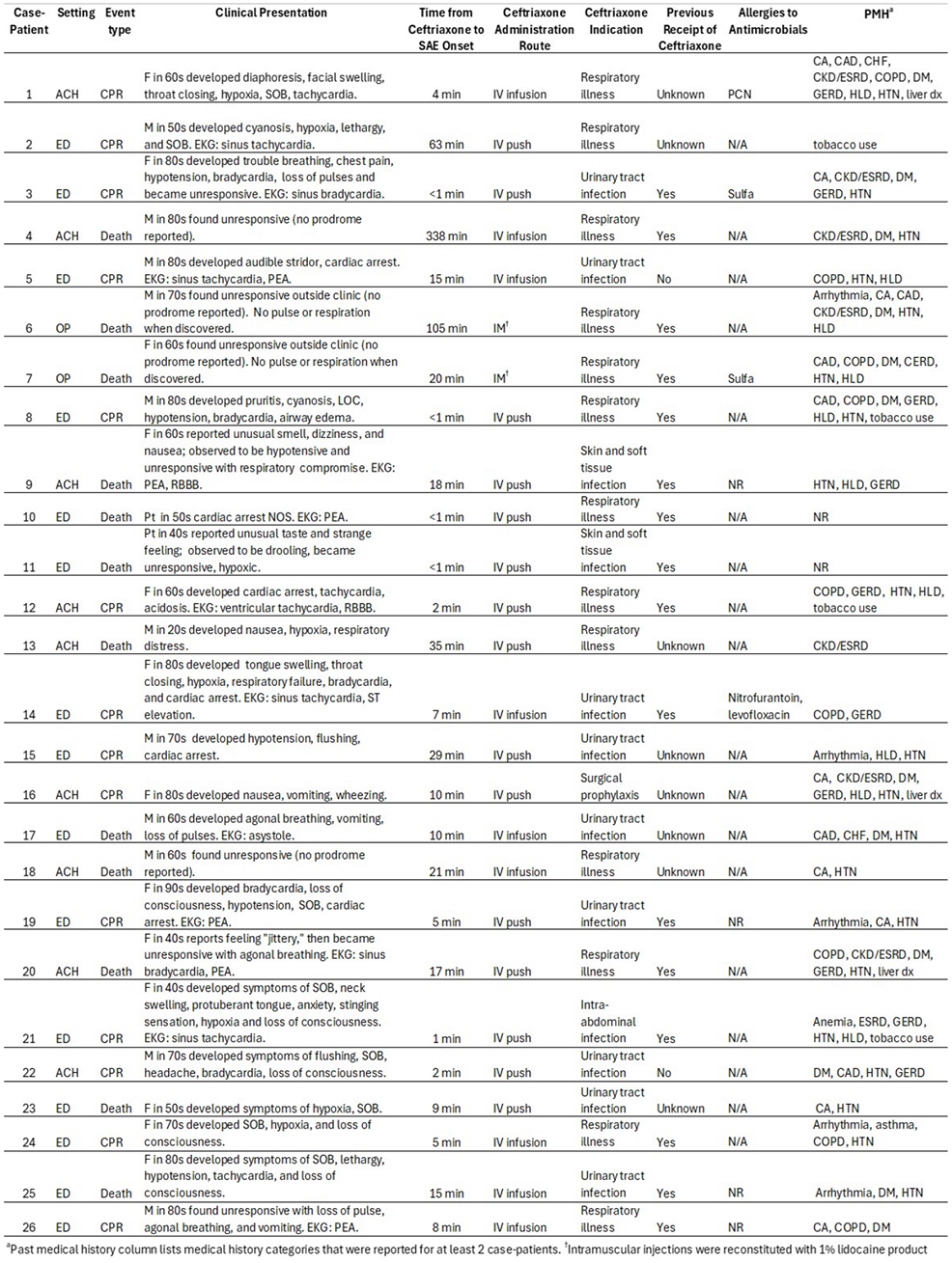
Clinical summary of serious adverse event cases (n=26), September 2024–June 2025 Abbreviations: SAE: serious adverse event, ACH: acute care hospital, ED: emergency department, OP: outpatient clinic, F: female, M: male, Pt: patient (used when sex not reported); NR: not reported, NOS: not otherwise specified, EKG: electrocardiogram, PEA: pulseless electrical activity, IV: intravenous, IM: intramuscular, N/A: not applicable, PMH: past medical history, CA: cancer, CAD: coronary artery disease, CHF: congestive heart failure, CKD/ESRD: chronic kidney disease or end stage renal disease; COPD: chronic obstructive pulmonary disease, DM: diabetes mellitus, HTN: hypertension, HLD: hyperlipidemia, dx: disease CPR: cardiopulmonary resuscitation.

**Figure 1a-b.**
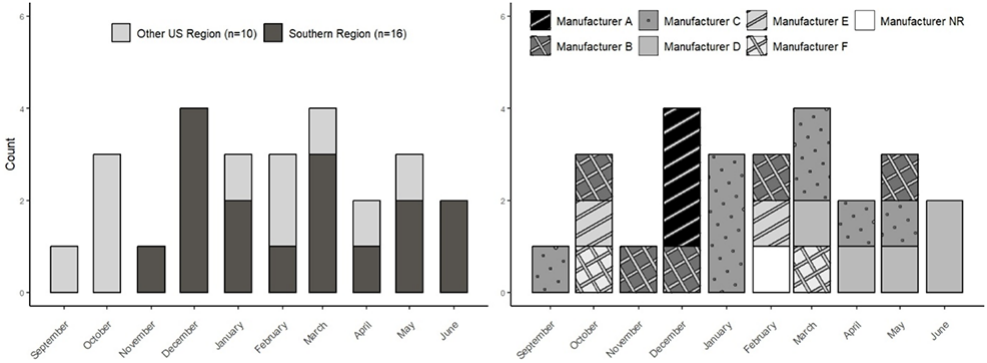
Epidemic curve: Frequency of reported serious adverse event cases following ceftriaxone administration from September 2024 to June 2025, by event month and US region (a) or ceftriaxone manufacturer (b), n=26 Figure includes SAEs meeting CDC case definition: Adverse events following injectable ceftriaxone exposure, occurring after September 1, 2024, which: 1) occurred within 6 hours after receipt of injectable ceftriaxone in a non-ICU setting, 2) resulted in death or required cardiopulmonary resuscitation (CPR), and 3) were not attributed by the treating provider(s) to a cause other than ceftriaxone administration (such as known infection, other underlying medical condition, or exposure to a medication or medical product other than ceftriaxone). Figure 1a (left) displays epidemic curve by US Census Bureau region, reflecting case-patient state of residence. Southern region includes AL, AR, DC, DE, FL, GA, KY, LA, MD, MS, NC, OK, SC, TN, TX, VA, WV. Figure 1b (right) displays epidemic curve by the ceftriaxone product reported as administered to case-patient or present in the facility at the time of adverse event. Abbreviations: NR: not reported, US: United States. (R Core Team, 2024).

Clinical summary of serious adverse event cases (n=26), September 2024–June 2025

Abbreviations: SAE: serious adverse event, ACH: acute care hospital, ED: emergency department, OP: outpatient clinic, F: female, M: male, Pt: patient (used when sex not reported); NR: not reported, NOS: not otherwise specified, EKG: electrocardiogram, PEA: pulseless electrical activity, IV: intravenous, IM: intramuscular, N/A: not applicable, PMH: past medical history, CA: cancer, CAD: coronary artery disease, CHF: congestive heart failure, CKD/ESRD: chronic kidney disease or end stage renal disease; COPD: chronic obstructive pulmonary disease, DM: diabetes mellitus, HTN: hypertension, HLD: hyperlipidemia, dx: disease CPR: cardiopulmonary resuscitation.

Epidemic curve: Frequency of reported serious adverse event cases following ceftriaxone administration from September 2024 to June 2025, by event month and US region (a) or ceftriaxone manufacturer (b), n=26

Figure includes SAEs meeting CDC case definition: Adverse events following injectable ceftriaxone exposure, occurring after September 1, 2024, which: 1) occurred within 6 hours after receipt of injectable ceftriaxone in a non-ICU setting, 2) resulted in death or required cardiopulmonary resuscitation (CPR), and 3) were not attributed by the treating provider(s) to a cause other than ceftriaxone administration (such as known infection, other underlying medical condition, or exposure to a medication or medical product other than ceftriaxone). Figure 1a (left) displays epidemic curve by US Census Bureau region, reflecting case-patient state of residence. Southern region includes AL, AR, DC, DE, FL, GA, KY, LA, MD, MS, NC, OK, SC, TN, TX, VA, WV. Figure 1b (right) displays epidemic curve by the ceftriaxone product reported as administered to case-patient or present in the facility at the time of adverse event. Abbreviations: NR: not reported, US: United States. (R Core Team, 2024).

**Methods**: CDC issued a national call for cases, defined as death or cardiopulmonary resuscitation within 6 hours of ceftriaxone receipt in non-intensive care settings without other apparent cause, occurring after Sep 1, 2024. Health departments collected clinical data for analysis at CDC. We further classified cases as anaphylaxis-type if determined by treating provider to be allergic or if involved two or more of: hypotension, respiratory compromise, cutaneous manifestations, and gastrointestinal symptoms. For SAEs reported Dec 2024-Jan 2025, FDA collected available ceftriaxone/diluent for testing and requested internal product investigations by manufacturers.

**Figure 2.**
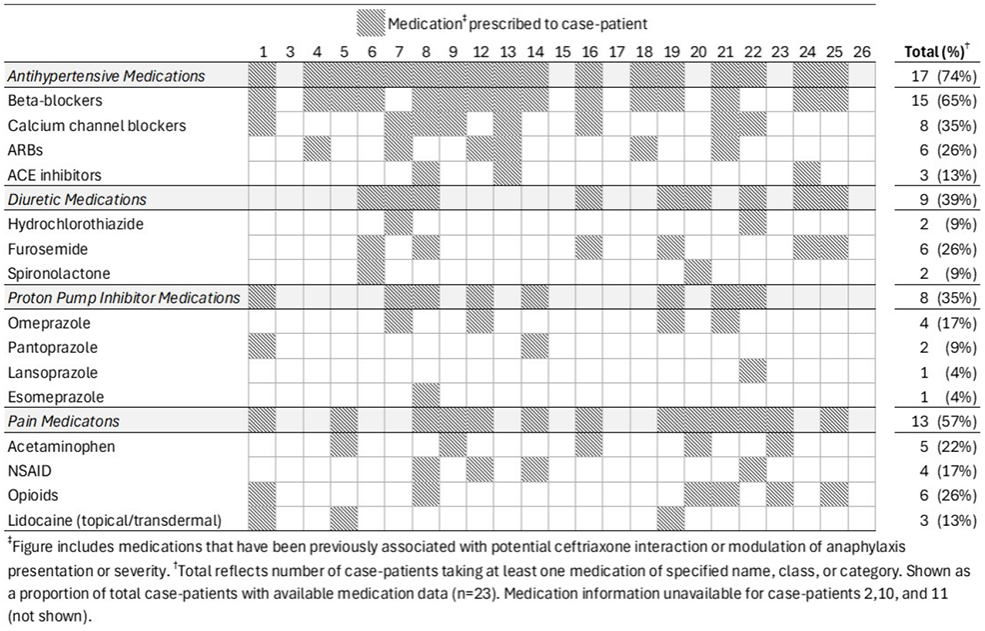
Medications (other than ceftriaxone) prescribed proximal and prior to serious adverse event, by individual case-patient, n=23 Abbreviations: ARB: angiotensin receptor blocker, ACE: angiotensin-converting enzyme, NSAID: non-steroidal anti-inflammatory drugs.

Medications (other than ceftriaxone) prescribed proximal and prior to serious adverse event, by individual case-patient, n=23

Abbreviations: ARB: angiotensin receptor blocker, ACE: angiotensin-converting enzyme, NSAID: non-steroidal anti-inflammatory drugs.

**Results**: We report 26 cases (65% outpatient; 35% inpatient), including 12 deaths, from 22 healthcare facilities across 15 states from Sep 1, 2024 to Jun 30, 2025 (Figure 1a). Case-patients had a median age of 70 years (IQR: 60-81) and received 1g (n=15, 58%), 2g (n=9, 35%), or unspecified (n=2, 7%) ceftriaxone doses via intravenous (IV) push (n=15, 58%), IV infusion (n=9, 35%), or intramuscular injection (n=2, 7%). Presentations varied (Table); 69% (n=18) were anaphylaxis-type. Prior ceftriaxone exposure (n=16, 62%), cardiac comorbidities (n=20, 77%), and/or concurrent use of antihypertensives (n=17, 65%, Figure 2) were common. Product exposures included 22 lots from 6 ceftriaxone manufacturers (Figure 1b). FDA testing of ceftriaxone (5 lots, 4 manufacturers) and lidocaine diluent (3 lots, 2 manufacturers) found no evidence of tampering, adulteration, endotoxin, or purity/potency issues. Manufacturers (n=4) did not report product anomalies.

**Conclusion**: SAEs, including deaths, can occur after ceftriaxone use. This investigation did not identify a common-source etiology, nor a link to a specific ceftriaxone product. Providers in all settings should monitor for SAEs, including anaphylaxis, and report to the FDA MedWatch Program.

**Disclosures**: All Authors: No reported disclosures

